# Final Year Nursing Students' Knowledge and Attitudes regarding Children's Pain

**DOI:** 10.1155/2020/7283473

**Published:** 2020-02-13

**Authors:** Abigail Kusi Amponsah, Joana Kyei-Dompim, Evans Frimpong Kyei, Evans Oduro, Richard Adongo Afaya, Collins Kwadwo Ahoto

**Affiliations:** ^1^Department of Nursing, Faculty of Allied Health Sciences, Kwame Nkrumah University of Science and Technology, Kumasi, Ghana; ^2^Department of Nursing Sciences, Faculty of Medicine, University of Turku, Turku, Finland; ^3^Asonomaso Government Hospital, Kwabre East, Ashanti, Ghana

## Abstract

Pain is one of the commonest reasons why children visit the hospital. Inadequately treated pain in children can negatively affect their physical, psychological, and social well-being; it also places financial burden on families of affected children and healthcare systems in general. Considering the eventual suffering of vulnerable children and their families if nursing students are insufficiently educated and ill-prepared, the current study aimed at assessing final year nursing student's knowledge and attitudes pertaining to pediatric pain. A descriptive cross-sectional study was conducted among 100 final year undergraduate nursing students at a private university college in Ghana. In addition to their ages and gender, the students responded to the 42 individual items on the Pediatric Nurses' Knowledge and Attitudes Survey regarding pain (PNKAS) instrument. Descriptive statistical analysis was aided by the Statistical Package for Social Sciences version 25 software. The mean age of the final year nursing students was 29 years (range of 21 to 47 years); a majority of them were females (78%). Participants had an average (SD) correct answer score of 44.0% (10.6%). Good pediatric pain knowledge and attitudes were observed in items that were related to the individualized and multidimensional nature of the pain experience and its treatment, benefits of pre-emptive analgesia, pharmacodynamics, and pain assessment. Poor pediatric pain knowledge and attitudes occurred in items that focused on pain perceptions, opioid drug administration, useful pain medications, pain physiology, and nonpharmacological pain management interventions. Final year nursing students have insufficient knowledge and attitudes toward children's pain management. Areas of good and poor pediatric pain knowledge and attitudes should be considered when designing and implementing educational interventions on this subject. Curricular revisions should be made on existing nursing curriculum to lay more emphasis on children's pain management and use educational interventions that support knowledge translation for improved care.

## 1. Introduction

Pain is one of the commonest reasons why children visit the hospital and a major source of discomfort for the children-in-pain, their families, and healthcare providers. Recent studies report a pain prevalence of 59%–94% among hospitalized children [1–3] with 27%–40% of them experiencing moderate-to-severe pain [3, 4]. The experience of pain among children may result from medical disorders, invasive procedures, physical injuries, and unknown factors [5, 6]. Inadequately treated pain does not only affect the physical growth and development of children but also their psychological well-being and social interactions with others [7, 8]. It also places a lot of burden on families of affected children and healthcare systems in general [9]. All of these negative consequences reinforce the urgent need for optimal pain treatment among vulnerable children.

Nurses are in a key position to rightfully assess and treat pediatric pain as they form majority of healthcare professionals and spend more contact hours interacting with children and their families during hospitalization. Unrelieved pediatric pain still persists as most healthcare professionals including nurses continue to exhibit inadequate knowledge and inappropriate attitudes toward its management [10–12]. Sufficient knowledge and positive attitudes of nurses and other healthcare professionals are thus required in improving the assessment and management of children's pain.

Nursing students generally go through a three- or four-year programme to prepare them for the world of nursing work [13]. As part of their educational preparation, they undertake clinical placements in children's units and may work in those units upon successful graduation. It is therefore important to assess their educational preparedness in assessing and managing children's pain. A review of recent literature revealed that unsatisfactory pediatric pain knowledge and attitudes have been reported among nursing students in Mexico [12] and Egypt [14].

It appears from the reviewed literature that there are a limited number of studies in this area. Considering the eventual suffering of vulnerable children and their families if nursing students are insufficiently educated and ill-prepared, the current study aimed at assessing final year nursing student's knowledge and attitudes pertaining to pediatric pain. The assessment of these students could also bring to light information that may assist in the development of appropriate strategies to address educational needs related to pediatric pain management.

## 2. Materials and Methods

### 2.1. Study Design, Setting, and Participants

A descriptive cross-sectional survey was conducted at a private university college in Ghana. This university college runs a four-year undergraduate general nursing programme which is operationalized through two main streams: regular and weekend group. The regular group attends lectures and practical sessions during the weekdays (Mondays to Fridays) whereas the weekend group operationalizes their activities from Fridays to Sundays. The weekend programme is mainly designed for nurses who already have Diploma qualifications and are in the process of upgrading them to Bachelor degrees. As part of the general nursing programme, students take courses in basic and advanced nursing, medicine, surgery, pediatrics, obstetrics and gynecology, public health, and mental health nursing. Upon examination of the undergraduate nursing curriculum of the university college, it was identified that the subject of “pain assessment and management” is part of the basic nursing course and not specifically outlined in their pediatric nursing course. The nursing students also undertake clinical placements in pediatrics during their third year of training.

The total number of final year nursing students was 117 as at the time of the study. The appropriate sample size for the study was estimated using Taro Yamane's formula [15]. A 95% confidence interval and a type I error-rate of 5% were used, resulting in a required number of 90.5. Considering a 10% nonresponse rate, an estimated sample size of 100 was considered sufficient in powering the present study.

### 2.2. Data Collection Procedures

After obtaining approval from the authorities of the university college and the ethics committee, the researchers approached the final year students during their free periods in between lectures and other school programs through their class leaders. The scope and objectives of the research were explained to them prior to the acquisition of their informed consent. During the month of October 2018, 103 final year nursing students were approached, out of which 100 gave their approval and participated in the present study, giving a response rate of 97%.

For anonymity and confidentiality purposes, participants provided their ages and gender on the data collection instrument without revealing their names. Over an average duration of 30 minutes, the final year nursing students responded to the 42 individual items on the Pediatric Nurses' Knowledge and Attitudes Survey regarding pain (PNKAS) instrument (Manworren, 2001). This survey instrument does not distinguish between knowledge and attitude items and measures the two constructs collectively.

The PNKAS instrument comprises 22 binary-response-type questions (true or false responses), 16 multiple choice questions (MCQs), and two case studies expanded into four MCQs. A score of one was given to a correctly answered knowledge and attitude question whilst a score of zero was provided for an incorrectly answered question. Thus, the minimum attainable score on the 42-item instrument was zero, and the maximum attainable score was 42. The total scores for each individual participant were converted into percentage using the formula: total percentage score = (total score obtained/42) *∗* 100.

Content validity of the PNKAS instrument has been previously established by five national pain experts (Manworren, 2001). An appreciable level of instrument reliability has been reported among 12 healthcare providers with a test-retest correlation coefficient of 0.67. An acceptable level of internal homogeneity has also been found among two separate groups of children's nurses with Cronbach's alpha coefficients of 0.72 and 0.77, respectively. Prior to the instrument's usage in the current study, its face validity was evaluated by 11 pediatric experts in Ghana.

### 2.3. Statistical Analysis

Data were first entered and cleaned on Microsoft Excel before being exported unto the Statistical Package for Social Sciences (SPSS) software version 25 for further analysis. Continuous variables were reported as means and standard deviations (SDs); categorical variables were also presented as frequencies and percentages.

### 2.4. Ethical Considerations

Administrative permission was obtained from the management of the university college prior to securing ethical approval from the Committee for Human Research, Publications and Ethics (CHRPE), School of Medical Sciences (SMS), Kwame Nkrumah University of Science and Technology (KNUST). After explaining the study protocol to the final year nursing students, the completion and submission of the questionnaire served as evidence for their informed consent. Participation in the current study was voluntary, and participants did not receive any penalties in situations where it was declined.

## 3. Results

### 3.1. Demographic Characteristics of Participants

The average (SD) age of the final year nursing students was 29 (5) years, ranging from 21 to 47 years; a majority of them were female (78%) (see Table 1).

### 3.2. Participants' Knowledge and Attitudes regarding Children's Pain

Participants' mean (SD) correct answer score on the PNKAS instrument was 44.0% (10.6%) and extended from a minimum score of 23.8% to a maximum of 85.7% (refer to Table 1). The rate of correct answers on all 42 items ranged from 2% to 81%.

As illustrated in Table 2, areas of good pediatric pain knowledge and attitudes centered on individualized and multidimensional nature of the pain experience and its treatment, benefits of pre-emptive analgesia, pharmacodynamics (effects of drugs on the body), and pain assessment.

The top 10 items which were frequently answered incorrectly were generally focused on pain perceptions, opioid drug administration, useful pain medications, pain physiology, and nonpharmacological pain management interventions (refer to Table 3).

## 4. Discussion

The present study aimed at assessing final year nursing students' knowledge and attitudes of pertaining to children's pain management at a private university college in Ghana. Our study results showed that the students had insufficient knowledge and attitudes toward children's pain management as the average score was less than 50%. This finding concurs with recent studies in Mexico [12] and Egypt [14] where they found poor pediatric pain knowledge and attitudes among the studied nursing students. The problem of insufficient pediatric pain knowledge and attitudes among nursing students is alarming and has implications for nursing education. It seems on the basis of this study and earlier studies that the prevailing nursing education is not adequately preparing nursing students in the area of children's pain management. This raises questions about the nature of education given to nursing students on pediatric pain assessment and management. Suffice to say that we are not adequately preparing nursing students to address the pain care needs of vulnerable children and their families.

In spite of the generally poor pediatric pain knowledge and attitudes, majority of the final year nursing students correctly answered items that were related to the individualized and multidimensional nature of the pain experience and its treatment, benefits of pre-emptive analgesia, pharmacodynamics, and pain assessment. Many of these areas of strength were similar to those identified in earlier studies [12, 14] and should be considered in the design and implementation of educational interventions on this subject. This further suggests that a greater proportion of the nursing students have good understanding about the subjective, complex, and multidimensional nature of the pain experience and its management [16]. They also appreciated the role of pre-emptive analgesia in preventing anticipated pain and promoting cooperation during procedures [17] and the therapeutic effects of analgesics on children [18]. Many of the sampled final year nursing students demonstrated sufficient knowledge and appropriate attitudes toward the adequacy of self-report in children less than 8 years of age. Studies have shown that children over five years can sufficiently describe the location and extent of their pain [19, 20]. While behavioral (such as facial expression, movement, crying, and consolability) and physiological signs (such as temperature, heart rate, and respiration) can be used in assessing pain among children with nonfunctional speech, their use should be limited among children who can effectively communicate as self-report which is the best measure of the subjective pain experience [21].

The present study also showed that the final year nursing students had poor knowledge and inappropriate attitudes toward pain assessment, opioid drug administration, useful pain medications, pain physiology, nonpharmacological pain management interventions, and pain perceptions. Many of these results are consistent with those identified by Ortiz and colleagues in Mexico [12]. Insufficient educational preparation coupled with the shortage of pain specialists in various nursing faculties and hospitals in Ghana [22] may also be responsible for the observed results. More emphasis should be placed on the identified deficient areas during the development and implementation of future educational interventions on pediatric pain management. Such educational offerings should employ strategies that enhance knowledge translation so as to improve pain care outcomes for affected children and their families. On the basis of the study findings, amendments should be made to existing nursing curricular during revisions to lay more emphasis on children's pain management for improved outcomes. Periodic training should be given to nurses following graduation to update them on the current, best pediatric pain assessment and management evidence for improved pediatric pain care.

The study findings also have implications for practicing nurses as they serve as role models and preceptors to nursing students. Education of nurses on the current best evidence for pediatric pain management should also be strengthened so that they can positively impart these unto the nursing students during mentoring. The knowledge and attitudes of nursing faculty members should also be explored in order to holistically address the issue as they facilitate teaching and learning experiences of the nursing students.

## 5. Strengths and Limitations

To the best of our knowledge, this study is the first of its kind in Ghana (a low middle-income country in West Africa) and provides important information regarding the current and desired educational needs of final year nursing students on pediatric pain management.

In spite of this, the study was not without some limitations which should be considered when interpreting the findings. The study used a nonprobability sampling approach in a single private university college, the results of which may not be a true representation of final year nursing students in Ghana. Future studies should include training colleges and government-owned institutions to enhance the generalizability of the findings.

## 6. Conclusions

Final year nursing students in a private university college in Ghana have insufficient knowledge and attitudes toward children's pain management. Areas of good and poor pediatric pain knowledge and attitudes should be considered when designing and implementing educational interventions on this subject. On the basis of these findings, amendments should be made to existing nursing curricular during revisions to lay more emphasis on children's pain management so as to improve pain care for vulnerable children and their families. Practicing nurses and faculty members should be supported so that they can positively impart the appropriate knowledge and attitudes to nursing students.

## Figures and Tables

**Table 1:** Participants' demographic characteristics and pediatric pain knowledge and attitude scores (*n* = 100).

Variable	Frequency (%)	Mean (SD)	Range
Age		29 (5)	21–47
Gender			
Male	22		
Female	78		
PNKAS (%)		44.0 (10.6)	23.8–85.7

Note: SD: standard deviation; PNKAS: Pediatric Nurses' Knowledge and Attitudes Survey regarding pain.

**Table 2:** Top 10 areas of pediatric pain knowledge and attitudes (*n* = 100).

Items (correct answer)	% correct
(1) After the initial recommended dose of opioid analgesic, subsequent doses should be adjusted in accordance with the individual patient's response (true)	81
(2) Comparable stimuli in different people produce the same intensity of pain (false)	78
(3) Children who will require repeated painful procedures should receive maximum treatment for the pain and anxiety of the first procedure to minimize the development of anticipatory anxiety before subsequent procedures (true)	75
(4) Combining analgesics and nondrug therapies that work by different mechanisms may result in better pain control with fewer side effects than using a single analgesic agent (true)	74
(5) Spiritual beliefs may lead a child to think that pain and suffering are necessary (true)	70
(6) Parents should not be present during painful procedures (false)	68
(7) Benzodiazepines do not reliably potentiate the analgesia of opioids unless the pain is related to muscle spasms (true)	67
(8) Respiratory depression rarely occurs in children who have been receiving stable doses of opioids over months (true)	63
(9) The nurse should rely on the parent's assessment of the child's pain intensity as children less than 8 years cannot reliably report pain intensity (false)	63
(10) Ibuprofen and other nonsteroidal anti-inflammatory agents are not effective analgesics for bone pain caused by metastases (false)	62

**Table 3:** Bottom 10 areas of pediatric pain knowledge and attitudes (*n* = 100).

Question (answer)	% incorrect
(1) Which of the following drugs are potentially useful for treatment of children's cancer pain? (all of the above)	98
(2) A postoperative 15-year-old boy who consistently report of moderate-to-severe pain despite smiling with his visitor has been prescribed “morphine IV 1–3 mg q1h PRN pain relief.” The appropriate action for the nurse to take at this time is to: (administer morphine 3 mg IV now)	92
(3) The likelihood of narcotic addiction in a child whose pain is being treated with opioid analgesics is: (<1%)	92
(4) A postoperative 15-year-old boy who consistently report of moderate-to-severe pain and grimaces upon turning in bed has been prescribed “morphine IV 1–3 mg q1h PRN pain relief.” The appropriate action for the nurse to take at this time is to: (administer morphine 3 mg IV now)	81
(5) Nondrug interventions are very effective for mild-moderate pain control but are rarely helpful for more severe pain (false)	81
(6) Observable changes in vital signs must be relied upon to verify a child's statement that he or she has severe pain (false)	81
(7) Your pain assessment of a postoperative 15-year-old boy who self-reports his pain as 8 despite smiling with his visitor should be: (8)	79
(8) The percentage of patients who over-report pain is: (0 or 10%)	77
(9) Anxiolytics, sedatives, and barbiturates are appropriate medications for the relief of pain during painful procedures (false)	72
(10) Giving children sterile water by injection (placebo) is often a useful test to determine if the pain is real (false)	72

Note: IV: intravenous, mg: milligram, q1h: hourly, and PRN: when necessary.

## Data Availability

Data supporting this work have been attached as supplementary information.

## Supplementary Materials

Supplementary Materials These data were collected during a descriptive cross-sectional survey to assess 100 final year nursing students' knowledge and attitudes regarding children's pain. The overall goal of this was to evaluate their educational preparedness on this subject as they would soon be joining the nursing workforce to take care of patients including hospitalized children and their families. The data consist of participants' identification number, age, and gender, as well as their pediatric pain knowledge and attitudes measured using the Pediatric Nurses' Knowledge and Attitudes regarding pain (PNKAS) instrument (Manworren, 2001). The 42-item PNKAS instrument consists of 25 binary-response-type questions (true or false), 13 multiple choice questions (MCQs), and two (2) case studies expanded into four (4) questions. A correctly answered question attracted a score of one (1) whereas a wrongly answered item was given a zero (0) score. Thus, the minimum attainable score on the PNKAS instrument is zero (0), and the maximum attainable score is 42. For standardization purposes, the total score obtained by each participant was converted into percentages using the formula (score obtained/42) *∗* 100. The data file (named PNKAS Data for Final Year Nursing Students KARPP IMPT Excel) consists of two (2) excel sheets: the first sheet contains the raw coded data, and the second sheet contains the code for variables found on the raw coded data.
